# Prevalence and Prognostic Significance of Restriction Versus Systolic Dysfunction in Patients With Transthyretin and Light Chain Cardiac Amyloidosis

**DOI:** 10.1161/CIRCHEARTFAILURE.125.012337

**Published:** 2026-02-24

**Authors:** Mattia Zampieri, Giulia Biagioni, Annamaria Del Franco, Marco Canepa, Italo Porto, Margherita Zanoletti, Marianna Eleonora Labate, Aldostefano Porcari, Luca Bordignon, Marco Merlo, Gianfranco Sinagra, Giacomo Tini, Beatrice Musumeci, Emanuele Barbato, Camillo Autore, Elena Biagini, Simone Longhi, Giuseppe Sena, Alberto Ponziani, Giulia Saturi, Vera Fico, Alessia Argirò, Carlotta Mazzoni, Carlo Fumagalli, Iacopo Olivotto, Federico Perfetto, Francesco Cappelli

**Affiliations:** 1 Department of Clinical and Experimental Medicine, Tuscan Regional Amyloidosis Centre, Careggi University Hospital, Florence, Italy (M. Zampieri, G.B., A.D.F., V.F., A.A., C.M., C.F., F.P., F.C.).; 2 Cardiomyopathy Unit, Careggi University Hospital, Florence, Italy (M. Zampieri, G.B., A.D.F., V.F., A.A., C.M., C.F., I.O., F.C.).; 3 Pediatric Cardiology, Meyer Children’s Hospital IRCCS, Florence, Italy (M. Zampieri, I.O.).; 4 Cardiology Unit, Ospedale Policlinico San Martino IRCCS, Genoa, Italy (M.C., I.P., M.E.L.).; 5 Department of Internal Medicine, University of Genova, Italy (M.C., I.P., M. Zanoletti).; 6 Cardiovascular Department, Centre for Diagnosis and Treatment of Cardiomyopathies, Azienda Sanitaria Universitaria Giuliano-Isontina (ASUGI), University of Trieste, Italy (A. Porcari, L.B., M.M., G. Sinagra).; 7 European Reference Network for rare, low prevalence and complex diseases of the heart (ERN GUARD-Heart) (A. Porcari, L.B., M.M., G. Sinagra, E. Biagini, S.L.).; 8 Division of Medicine, National Amyloidosis Centre, University College London, Royal Free Campus, United Kingdom (A. Porcari).; 9 Dipartimento di Medicina Clinica e Molecolare, Sapienza Università di Roma, Italy (G.T., B.M., E. Barbato).; 10 San Raffaele Cassino, Frosinone, Italy (C.A.).; 11 Cardiology Unit, Cardiac Thoracic and Vascular Department, IRCCS Azienda Ospedaliero- Universitaria di Bologna, Italy (E. Biagini, S.L., G. Sena, A. Ponziani, G. Saturi).

**Keywords:** amyloidosis, glomerular filtration rate, heart failure, phenotype, stroke volume

## Abstract

**BACKGROUND:**

The tenet of cardiac amyloidosis (CA) as a paradigm of heart failure with restrictive ventricular physiology and preserved systolic function has come under scrutiny. We aimed to evaluate the prevalence and clinical significance of left ventricular (LV) systolic dysfunction versus restriction in a large real-world cohort with CA, assessed at the time of diagnosis.

**METHODS:**

We retrospectively analyzed 540 TTR (transthyretin)-CA and 280 AL (light chain)-CA. Patients were divided into 3 LV phenotypes: (1) preserved LV function: LV ejection fraction >40% associated with grade I diastolic dysfunction; (2) restriction: LV ejection fraction >40% associated with grade II/III diastolic dysfunction; (3) systolic dysfunction: LV ejection fraction ≤40% irrespective of diastolic function. We analyzed the progression from preserved LV function towards the other 2 LV phenotypes and survival free from the composite end point of all-cause mortality and heart transplantation.

**RESULTS:**

In TTR-CA, the prevalence of preserved LV function was 32.0%, restriction was 56.1%, and systolic dysfunction was 11.9%. Among patients with preserved LV function, at the last evaluation, the conversion rate to restriction was 16.3% and to systolic dysfunction was 1.8%. The 3-year freedom from the composite end point was 75%, 61%, and 44%, respectively. In AL-CA, the prevalence of preserved LV function was 32.9%, restriction was 58.6%, and systolic dysfunction was 8.5%. Among patients with preserved LV function, at the last evaluation, the conversion rate to restriction was 12.9%, and to systolic dysfunction was none. The 3-year freedom from the composite end point was 46%, 32%, and 21%, respectively.

**CONCLUSIONS:**

Restriction was the most common presenting phenotype, while preserved LV function represented approximately one-third. The rate of progression from preserved LV function towards restriction was high, whereas it was limited towards systolic dysfunction. Although patients with preserved LV function presented the best event-free survival, considering all-cause mortality and heart transplantation, compared with restriction or systolic dysfunction, these phenotypes are not independent predictors of this composite end point.

**What is New?**

- This large multicenter study challenges the traditional view of cardiac amyloidosis as a uniform model of restrictive cardiomyopathy and heart failure with preserved systolic function.
- At diagnosis, restriction was confined to <60% of patients with transthyretin or light-chain cardiac amyloidosis, while the remainder had preserved left ventricular function without advanced diastolic dysfunction, or, in a minority, systolic dysfunction.
- Over time, a substantial proportion of patients with preserved left ventricular function progressed to restriction, whereas a minority progressed to systolic dysfunction.

**What Are the Clinical Implications?**

- There is a gradient in the composite end point of all-cause mortality or heart transplantation: patients with preserved left ventricular function had the best freedom from the composite end point, followed by those with restriction and then those with systolic dysfunction; however, these differences may reflect baseline clinical status rather than left ventricular phenotype per second.
- The absence of restriction or presence of systolic dysfunction should not exclude the diagnosis of cardiac amyloidosis, particularly in patients with unexplained heart failure.
- Recognition of phenotypic heterogeneity of cardiac amyloidosis is critical for timely diagnosis and appropriate risk stratification.

Cardiac amyloidosis (CA) is the prototype of infiltrative diseases, largely attributable to 2 leading etiologies: TTR (transthyretin) related and AL (light chain amyloidosis).

In both TTR-CA and AL-CA, amyloid infiltration of myocardial walls induces ventricular thickening and reduces the ventricular cavity, leading to a progressive increase in cardiac chamber stiffness. Thus, CA has classically been considered a model of heart failure with left ventricular (LV) preserved systolic function,^1,2^ and it has long been included among restrictive cardiomyopathies in international classifications.^3^ However, this concept has recently come under scrutiny, since overt restriction is often limited to advanced stages of disease, while LV systolic dysfunction may play an important role in CA-related symptoms.^4^

To date, the real-world prevalence and rate of progression to restriction versus systolic dysfunction in CA patients remains elusive. Likewise, the prognostic relevance of these functional LV phenotypes is unresolved. This represents a relevant gap in knowledge, and may hinder our understanding of risk stratification, indications for advanced therapies, as well as response to treatment.

In the present study, we thus investigated a large cohort of patients with TTR-CA and AL-CA, assessing the prevalence and clinical impact of restriction versus systolic dysfunction at presentation.

## Methods

This study presents data from a multicenter registry, established by a consortium of 5 Italian regional referral centers for CA (Bologna, Ospedale Sant’Orsola, IRCCS Azienda Ospedaliero-Universitaria di Bologna; Firenze, Tuscan Regional Amyloid Referral center, Azienda Ospedaliera Universitaria Careggi; Genova, IRCCS Ospedale Policlinico San Martino; Roma, Azienda Ospedaliera Universitaria Sant’Andrea; Trieste, Azienda Sanitaria Universitaria Giuliano-Isontina), which maintained a longitudinal database capturing clinical and outcomes data of patients under care.

The study was conducted according to the Declaration of Helsinki, and informed consent was obtained under the institutional review board policies of the relevant hospital administrations (Leading Center: Careggi University Hospital Ethics Committee - 22586_oss). The data that support the findings of this study are available from the corresponding author on reasonable request.

### Patient Selection and Diagnostic Criteria

All consecutive TTR-CA and AL-CA patients included in the registry from January 2000 to July 2022 with available clinical and echocardiographic follow-up information were enrolled in the present study (n=820).

Diagnosis of CA was made according to guidelines and recommendations contemporary to clinical practice.^5–10^ Diagnosis of AL amyloidosis was confirmed by biopsy of abdominal fat pad or of an involved organ with amyloid deposits and characterized as AL by immunohistochemistry, optic/immunoelectron microscopy, or proteomics. Before 2016, TTR-CA was diagnosed on the basis of suggestive cardiac imaging findings, and either direct endomyocardial biopsy or evidence of TTR amyloidosis in an extracardiac biopsy. After 2016, the diagnosis of both TTR-CA and AL-CA was established according to Gillmore’s Algorithm.^5^ All patients with TTR-CA, before and after 2016, underwent genetic sequencing of the TTR gene to distinguish variant TTR (TTRv)-CA from TTRwt (wild-type TTR)-CA.

### Echocardiographic Evaluation

As per standard protocol, patients underwent a comprehensive echocardiographic evaluation every 6 months. Echocardiographic evaluation was performed using conventional 2-dimensional and Doppler assessment. LV end-diastolic and end-systolic volumes and LV ejection fraction (EF) were calculated from apical views, using the Simpson biplane method.^11^

Single centers adjudicated the patterns of diastolic dysfunction at the time of echocardiographic evaluation, according to the American Society of Cardiology and internal protocols, and the degree of diastolic dysfunction was reported in a shared database as grade I or II/III.^12^ The grading of diastolic dysfunction in patients with atrial fibrillation was based on multiparametric assessment on several cardiac cycles at the time of evaluation and described in the final report as diastolic dysfunction with or without increased ventricular filling pressure. In the presence of atrial fibrillation and signs of increased ventricular filling pressure, diastolic function was adjudicated as grade II/III.

### Definitions

For the purpose of this study, 3 different patient groups were identified, based on LV phenotypes:

1. Preserved LV function: patients with LV-EF >40%^13^ and no restriction. The term preserved is used for clinical categorization, excluding those patients defined as reduced LV-EF or with grade II/III diastolic dysfunction,^12^ even though most patients with CA may show some degree of function impairment when assessed with more sensitive techniques than echocardiography.
2. Restriction: patients presenting with grade II/III diastolic dysfunction and concomitant LV-EF >40%. The term restriction is used to indicate an increased LV stiffness associated with abnormal filling pressure, which defines both grade II (pseudonormal pattern) and III (restrictive pattern) of diastolic dysfunction,^12^ since they have shown to portend similar poor outcomes in patients with heart failure.^14^
3. Systolic dysfunction: patients with reduced LV-EF ≤40%, regardless of diastolic function. This cutoff was adopted based on existing literature.^13^

According to existing literature patients with TTR-CA where also described based on National Amyloidosis Center (NAC) stages at diagnosis, defining 3 different groups as follows, stage 1: NT-proBNP (N-terminal pro-B-type natriuretic peptide) ≤3000 ng/L and estimated glomerular filtration rate ≥45 mL/min; stage 2: NT-proBNP >3000 ng/L or estimated glomerular filtration rate <45 mL/min; stage 3: NT-proBNP >3000 ng/L and estimated glomerular filtration rate <45 mL/min.^15^

Whereas patients with AL-CA where described based on European revised Mayo stage at diagnosis, this staging system uses a TnT cutoff level of 0.035 µg/L (or troponin I cutoff level of 0.10 µg/L or a high-sensitivity troponin T assay cutoff of 40 pg/mL) and NT-proBNP level of 332 ng/L to place patients with AL-CA into stage I: normal levels of both troponin and NT-proBNP; stage II: an elevated level of either but not both; stage III: elevated levels of both. Stage III is split into 2 subgroups depending on the absence (stage IIIA) or presence (stage IIIB) of an NT-proBNP level >8500 ng/L.^16^

### Follow-Up and Treatment Strategies

Patients underwent regular cardiological evaluations, including a comprehensive clinical assessment, ECG, and echocardiography, every 6 months, or more often, according to their clinical status. At each visit, pharmacological therapy was revised and modified according to the patients’ clinical status. Loop diuretics were evaluated and titrated based on HF symptoms and clinical findings.^17^

Currently, tafamidis is the only available disease-specific treatment for TTR-CA. However, its impact on our cohort was limited as the drug was approved by the Italian drug regulatory agency (Agenzia Italiana del Farmaco) at the end of October 2021 and became commercially available in 2022. At baseline, only 33 patients in the whole cohort initiated disease-modifying therapy—25 patients on tafamidis, and 8 on other TTR-specific agents (including patisiran, inotersen, acoramidis, or placebo as part of clinical trials).

All AL-CA patients were treated by hematologists with specific expertise in amyloidosis, according to national and international guidelines for hematological diseases.^18,19^

Over the years, treatment of AL has changed, and in the last 10 years, the main therapeutic regimen based on melphalan and dexamethasone has been gradually replaced by treatments based on bortezomib as cyclophosphamide, bortezomib, dexamethasone, or bortezomib, melphalan, dexamethasone.^20–22^ Finally, in 2021, the International Society of Amyloidosis and the European Hematology Association jointly published guidelines for patient-tailored treatment in AL.^18,19^ To evaluate hematological response to treatments at 6 months and 12 months, in AL-CA, we describe 4 possible haematological response: amyloid complete response: normal FLC (free light chains) ratio and negative serum and urine immunofixation; very good partial response: dFLC (difference between involved and uninvolved free light chains)<40 mg/L; partial response: dFLC decrease > 50%; and no response.^23^

### End Points

The end point of the study was a composite of all-cause mortality or heart transplantation, assessed across the 3 LV phenotypes (preserved LV function, restriction, and systolic dysfunction) as defined at diagnosis.

Moreover, the study aimed to explore the LV phenotypes progression and identify predictors of such progression.

### Statistical Analysis

Continuous variables were reported using the mean and SD, or median and interquartile range, depending on the distribution. The Shapiro-Wilk test was used to evaluate normality. χ^2^ or Fisher exact tests were used to compare categorical data, which were presented as frequencies and percentages.

As part of an ongoing prospective registry study, data from a retrospective analysis were examined. After establishing a priori 3 LV phenotype groups (preserved LV function, restriction and systolic dysfunction), we explored the effects of sex, age at diagnosis, New York Heart Association (NYHA) functional class, maximal septal wall thickness, dose of diuretic therapy, NAC stage (for TTR-CA), Mayo stage and treatment response (for AL-CA) on the likelihood of progression from preserved LV function to restriction or systolic dysfunction, both in AL-CA and TTR-CA, using competing-risk regression model according to Fine and Gray analysis and accounting for the composite end point as a competing event. The strength of the association between predictors and the occurrence of progression was expressed as a subhazard ratio with corresponding 95% CI. Missing values were excluded from the analysis.

To analyze whether the 3 LV phenotypes were associated with the composite end point, we performed Kaplan-Meier analysis with a log-rank test. Multivariable Cox regression models were then constructed to determine independent risk factors for the composite end point in both TTR-CA and AL-CA, and to test whether the 3 LV phenotypes were independently associated with outcome.

In line with domain expertise and prior literature, all variables deemed clinically relevant were included a priori in the multivariable Cox regression models. Specifically, the following covariates were entered into the models: sex, age at diagnosis, diabetes, hypertension, atrial fibrillation, NYHA functional class, maximal septal wall thickness, stroke volume index, LV phenotype at baseline, NAC stage (for TTR-CA), Mayo stage, haematological diagnosis, and hematologic response to treatment (for AL-CA). Multicollinearity was assessed by calculating the variance inflation factor, with a conservative threshold of 2.

All tests were 2-tailed, and a *P*≤0.05 was considered statistically significant. All analyses were conducted using R software, version 4.3.1 (R Foundation for Statistical Computing).

## Results

The study population included 540 patients with TTR-CA and 280 patients with AL-CA. Table S1 summarizes the clinical features at presentation for TTRwt-CA, TTRv-CA, and AL-CA. The main descriptive characteristics of the combined TTR-CA subgroups and AL-CA are listed below.

### TTR-CA

Of the 540 patients with TTR-CA, 458 (84.8%) were diagnosed as TTRwt-CA and 82 (15.2%) as TTRv-CA. Among those patients with TTRv-CA, the following genetic variants were identified: Ile88Leu, n=36 (43.9%); Val142Ile, n=14 (17.1%); Val50Met, n=10 (12.2%); Glu109Gln, n=7 (8.6%); Phe84Leu, n=5 (6.2%); Phe84Ile, n=2 (2.4%); Tyr98Phe, n=2 (2.4%); and Gly77Arg, n=1; Gly67Glu, n=1; Glu74Gln, n=1; Arg54Thr, n=1; Ala56Pro, n=1; Ala109Ser, n=1; representing 1.2% each.

**Figure 1.**
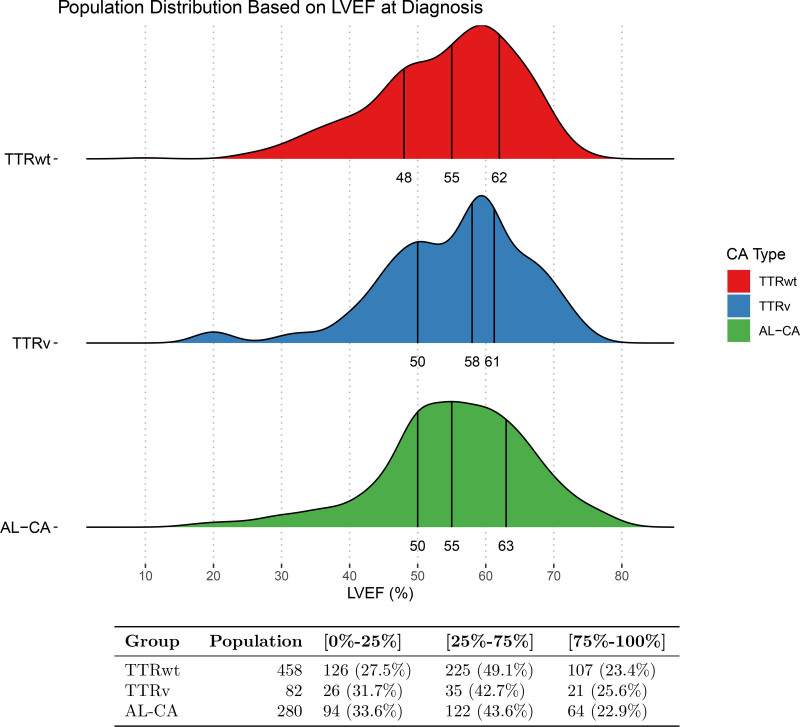
**Distribution of left ventricular ejection fraction (LV-EF) at diagnosis among wild-type transthyretin-related cardiac amyloidosis (TTRwt-CA), variant transthyretin-related cardiac amyloidosis (TTRv-CA), and light-chain cardiac amyloidosis (AL-CA).** Solid lines represent median values and interquartile ranges. The table reports the distribution of each population (number of patients and relative percentage) among the interquartile ranges 0% to 25%, 25% to 75%, and 75% to 100%.

Most patients with TTR-CA (n=482, 89.3%) were male, with a median age at diagnosis of 76 (interquartile range [IQR], 68–81) years. At first evaluation, 173 (32.0%) patients presented preserved LV function, 303 (56.1%) restriction, and 64 (11.9%) systolic dysfunction. Figure 1 illustrates the distribution of LV-EF among patients with TTR-CA. Most patients with preserved LV function presented NYHA class I or II, whereas most of the patients with restriction and systolic dysfunction presented NYHA II or III (Table). Median NTproBNP progressively increased from preserved LV function to restriction to systolic dysfunction, and similarly, NAC stage progressively worsened (Table).

**Table.**
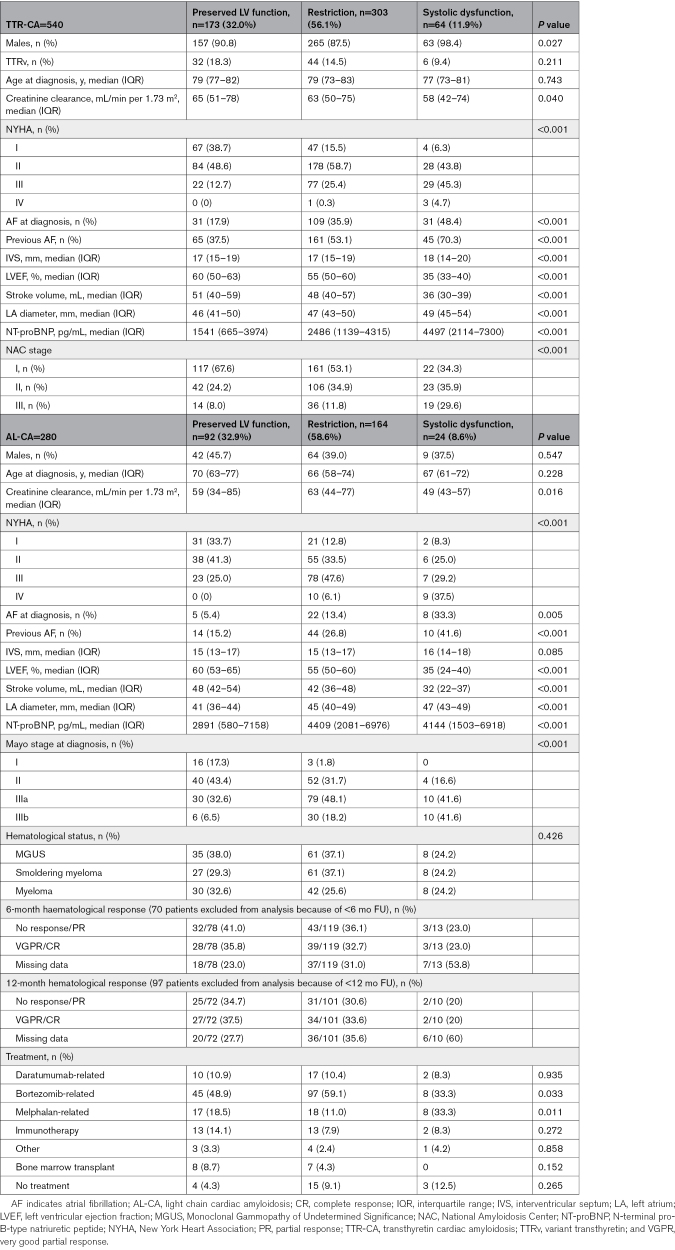
Demographic and Clinical Characteristics at Baseline According to the Left Ventricular Phenotypes in Patients With Transthyretin Cardiac Amyloidosis and Light Chain Cardiac Amyloidosis

### TTR-CA LV Phenotypes Progression

**Figure 2.**
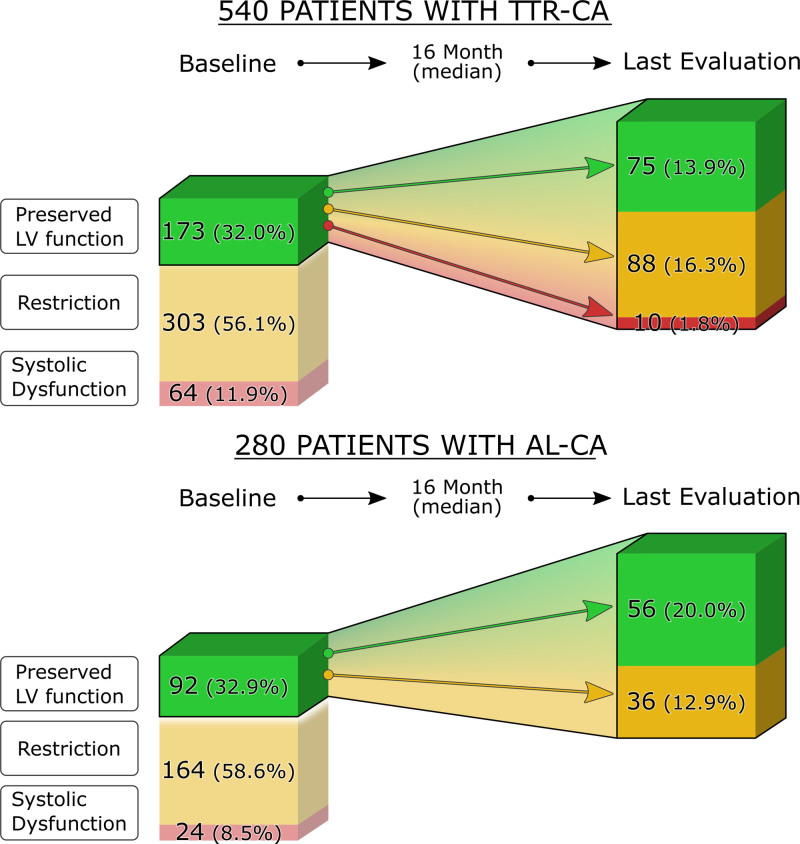
**Left ventricular (LV) phenotype progression in patients with transthyretin-related cardiac amyloidosis (TTR-CA) and light chain cardiac amyloidosis (AL-CA) with preserved left ventricular function at diagnosis.** Baseline categories are color-coded as follows: green, preserved LV function; yellow, restriction; and red, systolic dysfunction. Patient trajectory from preserved LV function is depicted as follows: green arrow, maintained preserved LV function; yellow arrow, progressed to restriction; and red arrow, progressed to systolic dysfunction.

At the last evaluation, after a median time of 16 months (7–31 months), 88 of the 173 patients with preserved LV function developed restriction, while 10 developed systolic dysfunction (Figure 2 shows baseline LV phenotypes and their progression at the last evaluation).

The 3-year conversion rate from preserved LV function to restriction was 63.4% and to systolic dysfunction 7.0% (Figure S1).

In the multivariable analysis, age at diagnosis (subhazard ratio, 1.04 [CI, 1.01–1.08]; *P*=0.017) and septal wall thickness (subhazard ratio, 1.13 [CI, 1.05–1.22]; *P*=0.001) were associated with the progression from preserved LV function to restriction or systolic dysfunction (Table S2).

### TTR-CA Composite End Point of All-Cause Mortality or Heart Transplantation

At 3 years follow-up, the composite end point was reached by 133/540 patients, among them 3 patients underwent heart transplant (3 patients with TTRwt-CA and 1 patient with TTRv-CA Ile68Leu).

**Figure 3.**
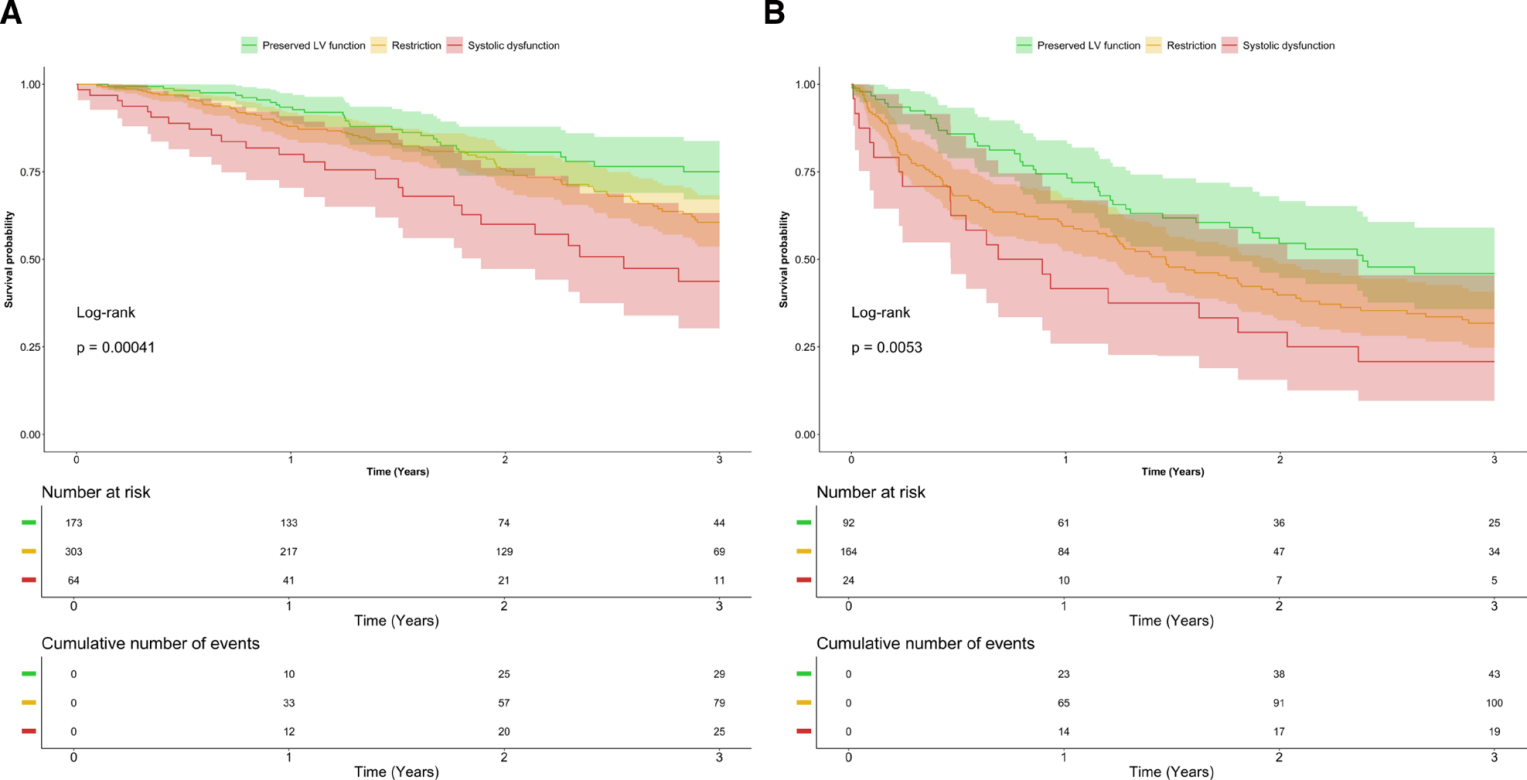
**Kaplan-Meier Survival curve for the composite end point of all-cause mortality or heart transplantation according to a discrimination among patients with preserved left ventricular (LV) function, restriction, and systolic dysfunction at presentation. A**, Patients with transthyretin-related cardiac amyloidosis (TTR-CA). **B**, Patients with light-chain cardiac amyloidosis (AL-CA).

Kaplan-Meier analysis in patients with TTR-CA (Figure 3A) demonstrated that freedom from the composite end point was 75% in those with preserved LV function, 61% in those with restriction, and 44% in those with systolic dysfunction (log-rank, *P*=0.0004).

In the multivariable Cox regression analysis, no interaction was found between LV phenotypes (preserved LV function versus restrictive or systolic dysfunction) and the composite end point; whereas, older age at diagnosis (hazard ratio [HR], 1.04 [CI, 1.01–1.08]; *P*=0.015), septal wall thickness (HR, 1.06 [CI, 1.01–1.13]; *P*=0.045), advanced NYHA class (NYHA III–IV versus NYHA I, HR, 2.83 [CI, 1.46–5.51]; *P*=0.002), and higher NAC stage (NAC II versus NAC I, HR, 1.73 [CI, 1.11–2.70]; *P*=0.015; NAC III versus NAC I, HR, 3.31 [CI, 1.97–5.54]; *P*<0.001) were associated with the composite end point (Table S3).

### AL-CA

Of the 280 patients with AL-CA, 160 (57.1%) patients were male, with a median age at diagnosis of 67.3 (IQR, 59.5–74.8) years. At diagnosis, 92 (32.9%) patients presented preserved LV function, 164 (58.6%) restriction, and 24 (8.5%) systolic dysfunction. Figure 1 illustrates the distribution of LV-EF among patients with AL-CA.

Median NT-proBNP was significantly different between patients with preserved LV function (2891 [IQR, 580–7158 pg/mL]) and patients with restriction (4409 [IQR, 2081–6976 pg/mL]) or systolic dysfunction (4144 [IQR, 1503–6918 pg/mL]), *P*<0.001.

Most patients with preserved LV function presented NYHA I or II, while most patients with restriction presented NYHA class II or III, and most patients with systolic dysfunction presented NYHA class III or IV (Table).

The European revised Mayo stage at diagnosis showed that the proportion of patients in stage IIIa/IIIb progressively increased from preserved LV function to restriction and systolic dysfunction (Table).

### AL-CA LV Phenotypes Progression

At the last evaluation, after a median follow-up of 16 months (7–30 months), 36 of the 92 patients with preserved LV function developed restriction, while none developed systolic dysfunction (Figure 2 shows baseline LV phenotypes and their progression at the last evaluation).

The 3-year conversion rate from preserved LV function to restriction was 43.8%, while none converted to systolic dysfunction (Figure S1).

In the multivariable Cox regression analysis, no independent predictors of progression from preserved LV function to restriction or systolic dysfunction were identified (Table S4).

### AL-CA Composite End Point of All-Cause Mortality or Heart Transplantation

At 3 years follow-up, the end point was reached by 162/280 patients, among them 2 patients underwent heart transplant.

Kaplan-Meier analysis in patients with AL-CA (Figure 3B) demonstrated that freedom from the composite end point was 46% in those with preserved LV function, 32% in those with restrictive physiology, and 21% in those with systolic dysfunction, log rank *P*=0.0053.

Multivariable Cox regression analysis showed no interaction between the LV phenotypes and the composite end point; whereas older age at diagnosis (HR, 1.03 [CI, 1.01–1.06]; *P*=0.003), advanced NYHA class (NYHA III–IV versus NYHA I: HR, 2.95 [CI, 1.32–6.63]; *P*=0.009), indexed stroke volume (HR, 0.95 [CI, 0.91–0.99]; *P*=0.011), Mayo stage (Mayo stage II versus I: HR, 3.81 [CI, 1.24–11.71]; *P*=0.020; Mayo stage IIIb versus I: HR, 6.54 [CI, 1.94–22.06]; *P*=0.002) and the underlying diagnosis of myeloma (myeloma versus Monoclonal Gammopathy of Undetermined Significance: HR, 2.06 [CI, 1.21–3.52]; *P*=0.008) were associated with the composite end point (Table S5).

## Discussion

**Figure 4.**
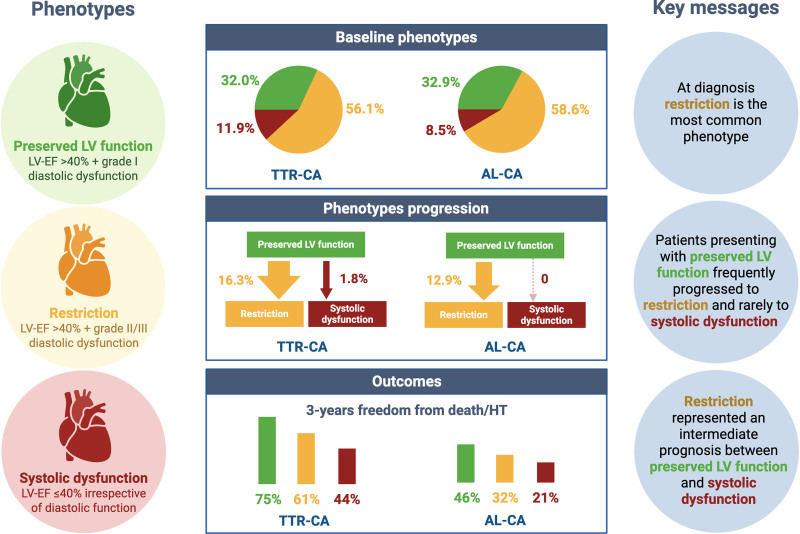
**Baseline distribution, longitudinal progression, and prognostic implications of LV phenotypes in cardiac amyloidosis.** The figure summarizes the prevalence of preserved LV function, restriction, and systolic dysfunction at diagnosis, their progression during follow-up, and their association with 3-year freedom from death or heart transplantation, demonstrating a stepwise deterioration in outcomes from preserved LV function to restriction and systolic dysfunction. AL-CA indicates light chain cardiac amyloidosis; LV-EF, left ventricular ejection fraction; and TTR-CA, transthyretin-related cardiac amyloidosis

This study, based on a large cohort of patients with TTR-CA and AL-CA referred to 5 Italian hub centers for amyloidosis, highlights the following key findings (Figure 4):

1. At diagnosis, restriction was the most common phenotype, while preserved LV function was present in one-third of patients, and ≈10% of the patients retained systolic dysfunction.
2. Patients presenting with preserved LV function frequently progressed to restriction and rarely to systolic dysfunction during follow-up. The 3-year rate of conversion from preserved LV function to restriction was 63.4% in TTR-CA and 43.8% in AL-CA (Figure 2).
3. TTR-CA or AL-CA with preserved LV function are those with the best freedom from the composite end point of all-cause mortality or heart transplantation, whereas restriction showed an intermediate prognosis between patients with preserved LV function and those with systolic dysfunction. However, these results may be attributable to underlying baseline differences between groups rather than the LV phenotype per second.

Restriction represented the most common phenotype (in TTR-CA, 56.1%; in AL-CA, 58.6%; Figure 2), constituting the absolute majority of patients with CA. Therefore, it is appropriate to define CA as a paradigm of restriction in less than half of patients, but this definition cannot be extended by default to the whole disease spectrum, which remains complex and heterogeneous. One-third of patients presented with preserved LV function (including all those patients with LV-EF >40% without diastolic dysfunction grade II/III). Thus, restriction is not a hallmark of CA, and its absence should not be a rule out criteria in the diagnostic process.

Similarly, CA can not be retained to the tenet of heart failure with preserved systolic function; we identified a wide range of LV-EF (Figure 1), with ≈1/10 of patients presenting with systolic dysfunction (LV-EF ≤40%), suggesting that reduced LV-EF can contribute to heart failure manifestations in CA. Thereafter, excluding patients with heart failure and systolic dysfunction from the possibility of an underlying CA is inaccurate and might result in diagnostic delay.

In our cohort, a significant proportion of patients, presenting with preserved LV function, progressed to restriction, whereas a minority progressed to systolic dysfunction (Figure 2). Indeed, an increase in myocardial amyloid deposition, quantified by using extracellular volume at cardiac magnetic resonance, has shown to be associated with progressive impairment of both diastolic (at lower extracellular volume values) and systolic function (at higher extracellular volume values).^24^ In TTR-CA, disease progression is likely the result of the long-standing myocardial amyloid deposition as a consequence of the prolonged absence of disease-modifying therapy. Whereas, in AL-CA, progression may be related to the direct cardiotoxic effect of free light chain,^25,26^ which can impair mitochondrial function,^25^ altering energy-dependent myocardial relaxation^27^ and lead to restriction even with a lower amount of amyloid deposition compared with TTR-CA.

Finally, as expected, CA was characterized by a high composite end point rate; however, the outcome showed different trends depending on the presenting LV phenotype. In TTR-CA and AL-CA, patients with preserved LV function were characterized by a lower rate of composite end point compared with patients with restriction or systolic dysfunction. Restriction represented an intermediate scenario between those with preserved LV function and those with systolic dysfunction, suggesting a gradient of severity (Figure 3A and 3B). Although other baseline features may also account for such differences in prognosis, the proposed paradigm appears reasonable from a pathophysiological point of view and may serve as a hypothesis-generating concept for future studies. In any case, based on our findings, the presence of restriction as a presenting phenotype should not be used to discriminate access for disease-modifying treatments.

Hopefully, disease-modifying therapy will profoundly change the natural history of both TTR-CA and AL-CA in the near future, calling for a revisitation of these findings in the coming years.

In our TTR-CA cohort, the role of disease-modifying therapies had a limited impact on prognosis, due to the small number of treated subjects and short treatment exposure. Thus, the present data assume an important value in describing the natural history of TTR-CA and provide a useful benchmark for evaluating the efficacy of developing therapies.

Conversely, patients with AL-CA represent a heterogeneous group who have received different chemotherapy regimens over the years. More aggressive treatment strategies were implemented in patients with preserved LV function, thought to better tolerate potential cardiotoxic effects. This represents an additional confounding factor in the comparison of different LV phenotypes; however reflects real-world strategies adopted in our centers.

### Limitations

This study has limitations, primarily related to its retrospective, observational design.

We focused on all-cause mortality and heart transplantation as study composite end points, no other end point was included (eg, heart failure hospitalization), and no granular evaluation on the cause of death was practical to describe.

Additionally, detailed information on hematologic response was not systematically collected for all patients, and it was not feasible to assess its impact on the composite end point.

### Conclusions

We reported substantial deviations from what has been commonly described as the classic CA phenotype, often defined as a paradigm of restriction or as heart failure with preserved systolic function. At the time of diagnosis, restriction was the most frequent LV phenotype in our CA population, affecting just under 60% of patients. Preserved LV function—defined by grade I diastolic dysfunction in the absence of systolic dysfunction—was present in roughly one third, whereas systolic dysfunction was detected in 10%.

Over time, patients presenting with preserved LV function frequently progress to restriction and, to a lesser extent, to systolic dysfunction. Although we observed a worsening trend in the composite end point across the 3 LV phenotypes, other baseline characteristics may account for this finding. Therefore, this classification can not currently be used to discriminate access to novel treatments.

## Article Information

### Sources of Funding

The work reported in this publication was partially funded by the Italian Ministry of Health, RC-2022-2773270 project.

### Disclosures

Dr Olivotto reported receiving research grants and personal fees from Cytokinetics, BMS, Tenaya, Lexeo, Rocket Pharma, Edgewise, and Sanofi Genzyme and grants from Menarini International, Amicus, and Chiesi. Dr Cappelli received honoraria from Pfizer, Alnylam, Novonordisk, Bayer, and Astra Zeneca bridgebio. Dr Perfetto has received honoraria for advisory board participation from Pfizer, Alnylam, and Akcea. Dr Biagini received advisory board fees from Sanofi, Genzyme, and Takeda. Dr Longhi is supported by the Italian Ministry of Health, RC-2024-2789983 project, and the European Reference Network for Rare, Low Prevalence and Complex Diseases of the Heart-ERN GUARD-Heart. Dr Sinagra received personal fees for occasional educational activities from Biotronik, Boston Scientific, AstraZeneca, and Novartis. Dr Canepa received speaker and advisor fees from Akcea Therapeutics, Menarini, Novartis, Pfizer, Sanofi e Sanofi Genzyme, and Vifor Pharma, as well as 2 investigator-initiated grants from Pfizer.

Dr Merlo received congress fees from Novartis and Vifor Pharma and a research grant and congress fees from Pfizer. The other authors report no conflicts.

### Supplemental Material

Table S1–S5

Figure S1

## Supplementary Material
