# Supplementary material for: Prevalence and Prognostic Significance of Restriction Versus Systolic Dysfunction in Patients With Transthyretin and Light Chain Cardiac Amyloidosis
Source: Circ Heart Fail. 2026 Feb 24;19(3):e012337. doi: 10.1161/CIRCHEARTFAILURE.125.012337 (PMC12986034; doi:10.1161/CIRCHEARTFAILURE.125.012337)
Supplement: Supplementary file 1 [file hhf-19-e012337-s001.pdf]

SUPPLEMENTAL MATERIAL

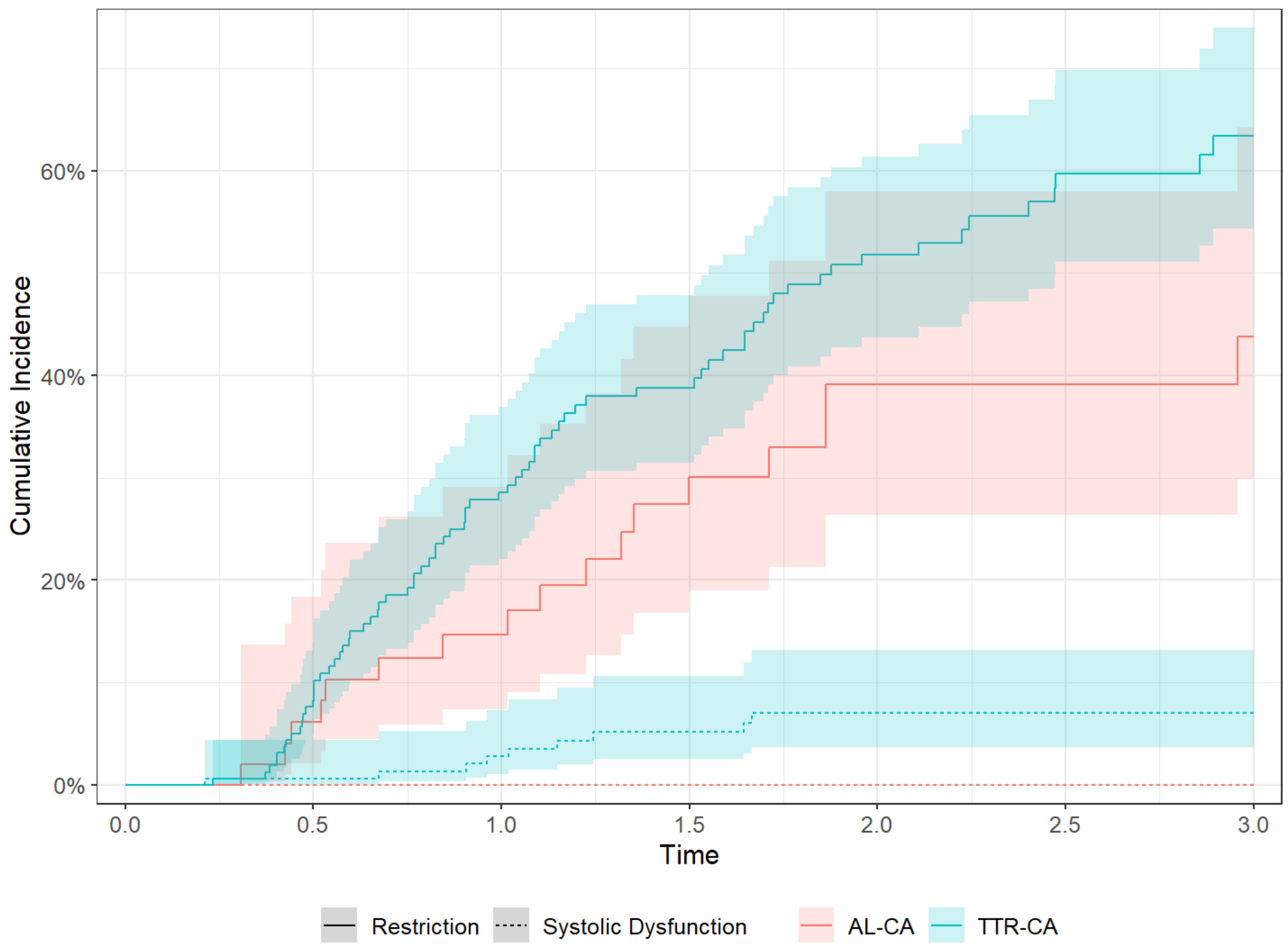


**Figure S1**: The 3-year conversion rate from *preserved LV function* to *restriction* or *systolic dysfunction* in patients with transthyretin cardiac amyloidosis (TTR-CA) and light-chain cardiac amyloidosis (AL-CA). Cumulative incidence curves are stratified by amyloidosis subtype (TTR-CA in green, AL-CA in red) and type of functional decline (*restriction* in solid lines; *systolic dysfunction* in dotted lines). Shaded areas represent 95% confidence intervals.

**Table S1.** Demographic and clinical characteristics at baseline in the initial cohort of patients with transthyretin cardiac amyloidosis (wild-type and hereditary) and light chain cardiac amyloidosis.

| | TTRwt-CA<br>n = 458 | TTRv-CA<br>n = 82 | AL-CA<br>n = 280 |
|---|---|---|---|
| Males, n (%) | 419 (91.4) | 63 (77.0) | 160 (57.1) |
| Caucasian, n (%)<br>Afro-Caribbean, n (%) | 458 (100)<br>0 | 81 (98.7)<br>1 (1.2) | 280 (100)<br>0 |
| BMI (Kg/$m^2$), median (IQR) | 25.3 (23.4-27.5) | 24.5 (21.5-27.5) | 24.0 (21.6-26.5) |
| Age at diagnosis (y), median (IQR) | 80 (74-83) | 71 (64-77) | 67 (59-75) |
| CAD, n (%) | 81 (17.7) | 11 (13.4) | 31 (11.1) |
| Diabetes, n (%) | 79 (17.2) | 8 (9.7) | 31 (11.1) |
| Hypertension, n (%) | 316 (69.0) | 40 (48.8) | 113 (40.4) |
| Creatinine Clearance (ml/min/1.73 $m^2$), median (IQR) | 55 (40-69) | 64 (48-81) | 57 (37-78) |
| NYHA, n (%)<br>I<br>II<br>III<br>IV | <br>95 (20.7)<br>246 (53.7)<br>113 (24.7)<br>4 (0.9) | <br>26 (31.7)<br>42 (51.2)<br>14 (17.1)<br>0 | <br>53 (18.9)<br>97 (34.7)<br>106 (37.8)<br>24 (8.6) |
| IVS (mm), median (IQR) | 17 (16-20) | 17 (15-20) | 15 (13-18) |
| EDD (mm), median (IQR) | 45 (41-49) | 42 (40-46) | 43 (39-47) |
| LVEF (%), median (IQR) | 50 (42-60) | 55 (45-60) | 57 (50-60) |
| LA diameter (mm), median (IQR) | 47 (44-50) | 46 (42-50) | 44 (40-48) |
| Diastolic dysfunction grade, n (%)<br>I<br>II/III | <br>152 (33.2)<br>306 (66.8) | <br>33 (40.2)<br>49 (59.8) | <br>96 (34.2)<br>184 (65.8) |
| NT-proBNP (pg/ml), median (IQR) | 2847 (1103-4966) | 1219 (496-3184) | 4081 (1862-8834) |

AL-CA: light chain cardiac amyloidosis, BMI: body mass index, CAD: coronary artery disease, EDD: end diastolic volume, IVS: interventricular septum, LA: left atrium, LVEF: left ventricular ejection fraction, NT-proBNP: NT-pro brain natriuretic peptide NYHA: New York Heart Association; TTRv-CA: hereditary transthyretin cardiac amyloidosis, TTRwt-CA: transthyretin wild-type cardiac amyloidosis.

Supplementary Table 2. Multivariable competing risk regression analysis for predictors of progression from preserved left ventricular function to restriction or systolic dysfunction in patients with transthyretin cardiac amyloidosis (TTR-CA). Results are expressed as subdistribution hazard ratios (SHR) with 95% confidence intervals (CI), derived from the Fine–Gray competing risks model.

*Multivariable Competing Risk Analysis in TTR-CA patients*

| **Variable** | **SHR** | **CI (95%)** | **P value** |
|---|---|---|---|
| **Gender (Male)** | | | |
| Female | 1.41 | 0.59-3.35 | 0.441 |
| **Age at Diagnosis** | 1.04 | 1.01-1.08 | 0.017 * |
| **TTR variant** | 1.88 | 0.83-4.24 | 0.128 |
| **NYHA Class (I)** | | | |
| II | 0.89 | 0.54-1.49 | 0.664 |
| III-IV | 0.79 | 0.33-1.90 | 0.600 |
| **Septal Wall Thickness** | 1.13 | 1.05-1.22 | 0.001 ** |
| **Diuretic Dosage (No diuretics)** | | | |
| Furosemide ≤ 125 mg/day | 1.16 | 0.65-2.05 | 0.621 |
| Furosemide > 125 mg/day | 0.80 | 0.11-5.60 | 0.819 |
| **NAC Stage (I)** | | | |
| II | 1.10 | 0.64-1.88 | 0.732 |
| III | 0.87 | 0.36-2.14 | 0.769 |
| Likelihood ratio test = 0.148 | | | |
| Wald test = 0.010 | | | |
| Score (logrank) test = 0.155 | | | |

Supplementary Table 3. Multivariable Cox proportional hazards regression analysis for predictors of the composite endpoint of all-cause mortality or heart transplantation in patients with transthyretin cardiac amyloidosis (TTR-CA). Results are expressed as hazard ratios (HR) with 95% confidence intervals (CI).

*Composite Endpoint in TTR-CA Patients- Multivariable Cox Regression*

| **Variable** | **HR** | **CI (95%)** | **P value** |
|---|---|---|---|
| **Gender (Male)** | | | |
| Female | 0.75 | 0.39-1.44 | 0.387 |
| **Age at Diagnosis** | 1.04 | 1.01-1.08 | 0.015 * |
| **Arterial Hypertension** | 0.78 | 0.53-1.15 | 0.210 |
| **Diabetes Mellitus** | 0.77 | 0.48-1.26 | 0.307 |
| **AF History (No)** | | | |
| Paroxysmal/persistent | 0.68 | 0.41-1.14 | 0.145 |
| Permanent | 0.99 | 0.64-1.52 | 0.959 |
| **TTR Variant** | 0.93 | 0.54-1.62 | 0.810 |
| **NYHA Class (I)** | | | |
| II | 1.34 | 0.72-2.50 | 0.356 |
| III-IV | 2.83 | 1.46-5.51 | 0.002 ** |
| **Septal Wall Thickness** | 1.06 | 1.01-1.13 | 0.045 * |
| **LV Phenotype at Diagnosis (Preserved LV function)** | | | |
| Restriction | 1.41 | 0.81-2.45 | 0.228 |
| Systolic dysfunction | 1.44 | 0.81-2.57 | 0.214 |
| **SVi** | 0.97 | 0.94-1.00 | 0.095 |
| **NAC Stage (I)** | | | |
| II | 1.73 | 1.11-2.70 | 0.015 * |
| III | 3.31 | 1.97-5.54 | <0.001 *** |
| Likelihood ratio test = <0.001 | | | |
| Wald test = <0.001 | | | |
| Score (logrank) test = <0.001 | | | |

Supplementary Table 4. Multivariable competing risk regression analysis for predictors of progression from preserved left ventricular function to restriction or systolic dysfunction in patients with light-chain cardiac amyloidosis (AL-CA). Results are expressed as subdistribution hazard ratios (SHR) with 95% confidence intervals (CI), derived from the Fine–Gray competing risks model.

*Multivariable Competing Risk Analysis in AL-CA patients*

| **Variable** | **SHR** | **CI (95%)** | **P value** |
|---|---|---|---|
| **Gender (Male)** | | | |
| Female | 0.86 | 0.17-4.55 | 0.875 |
| **Age at Diagnosis** | 1.05 | 0.94-1.56 | 0.349 |
| **NYHA Class (I)** | | | |
| II | 1.93 | 0.13-28.07 | 0.629 |
| III-IV | 2.09 | 0.15-30.00 | 0.586 |
| **Septal Wall Thickness** | 1.08 | 0.75-1.57 | 0.680 |
| **Diuretic Dosage (No diuretics)** | | | |
| Furosemide ≤ 125 mg/day | 1.27 | 0.28-5.69 | 0.751 |
| Furosemide > 125 mg/day | NA | NA | NA |
| **Mayo Stage (I)** | | | |
| II | 7.90 | 0.10-626.82 | 0.354 |
| III | 11.14 | 0.20-629.06 | 0.241 |
| **AL Treatment Response (within 12m) (No/partial response)** | | | |
| VGPR/complete response | 1.78 | 0.57-5.51 | 0.318 |
| **Haematological Diagnosis (MGUS)** | | | |
| Myeloma | 1.42 | 0.27-7.50 | 0.677 |
| Smouldering myeloma | 3.10 | 0.49-19.65 | 0.231 |
| Likelihood ratio test = 0.155 | | | |
| Wald test = <0.001 | | | |
| Score (logrank) test = 0.100 | | | |
| | | | |

Supplementary Table 5. Multivariable Cox proportional hazards regression analysis for predictors of the composite endpoint of all-cause mortality or heart transplantation in patients with light-chain cardiac amyloidosis (AL-CA). Results are expressed as hazard ratios (HR) with 95% confidence intervals (CI).

*Composite Endpoint in AL-CA Patients - Multivariable Cox Regression*

| Variable | HR | CI (95%) | P value |
|---|---|---|---|
| **Gender (Male)** | | | |
| Female | 1.065 | 0.66-1.72 | 0.795 |
| **Age at Diagnosis** | 1.03 | 1.01-1.06 | 0.003 ** |
| **Arterial Hypertension** | 0.90 | 0.59-1.38 | 0.624 |
| **Diabetes Mellitus** | 0.86 | 0.47-1.59 | 0.636 |
| **AF History (No)** | | | |
| Paroxysmal/persistent | 0.95 | 0.54-1.65 | 0.849 |
| Permanent | 0.87 | 0.37-2.04 | 0.743 |
| **NYHA Class (I)** | | | |
| II | 1.61 | 0.75-3.48 | 0.222 |
| III-IV | 2.95 | 1.32-6.63 | 0.009 ** |
| **Septal Wall Thickness** | 1.00 | 0.91-1.10 | 0.975 |
| **LV Phenotype Function at Diagnosis (Preserved LV function)** | | | |
| Restriction | 0.98 | 0.56-1.72 | 0.946 |
| Systolic dysfunction | 0.90 | 0.47-1.71 | 0.752 |
| **SVi** | 0.95 | 0.91-0.99 | 0.011 * |
| **Haematological Diagnosis (MGUS)** | | | |
| Myeloma | 2.06 | 1.21-3.52 | 0.008 ** |
| Smouldering myeloma | 0.87 | 0.54-1.41 | 0.581 |
| **Mayo Stage (I)** | | | |
| II | 3.81 | 1.24-11.71 | 0.020 * |
| IIIa | 2.84 | 0.90-8.96 | 0.074 |
| IIIb | 6.54 | 1.94-22.06 | 0.002 ** |
| Likelihood ratio test = <0.001 | | | |

| Variable | HR | CI (95%) | P value |
|---|---|---|---|
| Wald test = <0.001 | | | |
| Score (logrank) test = <0.001 | | | |

The overall goodness of fit of the model and the statistical significance of the covariates were assessed using the Likelihood Ratio test, Wald test, and Score (log-rank) test.
